# Future perspectives for PDE5 inhibitors bridging the gap between cardiovascular health and psychological status

**DOI:** 10.1186/s12610-024-00245-y

**Published:** 2025-01-27

**Authors:** Andrea Sansone, Eugenia Guida, Susanna Dolci, Valeria Frangione, Amanda Asso, Gilberto Bellia, Emmanuele A. Jannini

**Affiliations:** 1 Chair of Endocrinology and Medical Sexology (ENDOSEX), Dept. of Systems Medicine, University of Rome Tor Vergata, via Montpellier 1, Rome, 00133 Italy; 2 Chair of Anatomy, Dept. of Biomedicine and Prevention, University of Rome Tor Vergata, Rome, Italy; 3 IBSA Institut Biochimique SA, Pambio-Noranco, Switzerland; 4 IBSA Farmaceutici Italia S.r.l, Lodi, Italy

**Keywords:** Cardiovascular disease, Erectile disfunction, Oral Film, PDE5i, Sildenafil, Maladie cardiovasculaire, Dysfonction érectile, Film buccal, PDE5i, Sildénafil

## Abstract

The serendipitous discovery that inhibiting type 5 phosphodiesterase (PDE5) using sildenafil, a potent PDE5 inhibitor (PDE5i) initially developed for cardioprotection, introduced the possibility of orally managing erectile dysfunction (ED) led to an increase in research data, which are currently considered groundbreaking for the new discipline of sexual medicine. Findings from a number of laboratories and clinics around the world unanimously demonstrated the following: (i) the major cause of ED is directly or indirectly related to cardiovascular disease (CVD); (ii) ED and CVDs share the same risk factors, which are related mainly to lifestyle choices; (iii) the first therapeutic approach to both ED and CVDs is to transform harmful lifestyles into virtuous lifestyles; and (iv) PDE5is in general, particularly sildenafil, are very safe, if not protective, for use in CVD patients. However, the use of PDE5is has faced several challenges. Many patients and some healthcare providers (HCPs) often share the misconception that using these drugs can increase the risk of CVD. Some patients might desire to fulfill the unmet need for privacy linked to the stigma of being treated for ED or might be enticed by the idea of buying drugs online, either because of shame or cheaper prices, without knowing the risks associated with counterfeit drugs. The aim of this narrative revision of the current literature is to demonstrate that (i) the orodispersible film of sildenafil is safe from a CV perspective; (ii) it is a discreet formulation that respects the need for privacy; and (iii) it is virtually the unique PDE5i formulation too expensive to produce outside the correct channels, making it impossible to be counterfeit.

## Résumé

La découverte fortuite de l’inhibition de la phosphodiestérase de type 5 (PDE5) par l’utilisation du sildénafil, un puissant inhibiteur de la PDE5 (PDE5i), initialement développé pour la cardioprotection, a introduit la possibilité de gérer par voie orale la dysfonction érectile (DE) ; cela a conduit à une augmentation des données de recherche, qui sont actuellement considérées comme révolutionnaires pour la nouvelle discipline de la médecine sexuelle.

Les résultats d’un certain nombre de laboratoires et de cliniques à travers le monde ont montré à l’unanimité ce qui suit : i) la principale cause de la dysfonction érectile est directement ou indirectement liée aux maladies cardiovasculaires (MCV) ; ii) les troubles de l’érection et les maladies cardiovasculaires partagent les mêmes facteurs de risque, qui sont principalement liés aux choix de mode de vie ; iii) la première approche thérapeutique à la fois des troubles de l’érection et des maladies cardiovasculaires consiste à transformer les modes de vie nocifs en modes de vie vertueux ; et iv) les PDE5 en général, en particulier le sildénafil, sont très sûrs, sinon protecteurs, pour une utilisation chez les patients atteints de MCV.

Cependant, l’utilisation de PDE5is a été confrontée à plusieurs défis. De nombreux patients et certains professionnels de santé partagent souvent l’idée fausse que l’utilisation de ces médicaments peut augmenter le risque de MCV. Certains patients pourraient souhaiter combler le besoin non satisfait de confidentialité lié à la stigmatisation d’être traité pour une dysfonction érectile, ou pourraient être attirés par l’idée d’acheter des médicaments en ligne, soit par honte, soit à des prix moins chers, sans connaître les risques associés aux médicaments contrefaits.

L’objectif de cette revue narrative de la littérature actuelle est de démontrer que i) le film orodispersible du sildénafil est sans danger du point de vue du CV ; ii) il s’agit d’une formulation discrète qui respecte le besoin d’intimité ; et iii) il s’agit pratiquement de la formulation unique de la PDE5i trop chère pour être produite en dehors des canaux appropriés, ce qui rend impossible la contrefaçon.

## Introduction

Sildenafil citrate, the first oral drug proposed for the treatment of erectile dysfunction (ED), rapidly emerged as one of the world’s most renowned medications following its introduction. Its efficacy has attracted a significant amount of media attention, making the name Viagra extremely popular, to the point that it has been speculated that it was the fastest selling drug and the most famous brand name of all time [1]. Sildenafil exerts its action by inhibiting the enzyme phosphodiesterase type 5 (PDE5), which, while ubiquitous in the body, is mostly expressed in the penile cavernous tissue. In other words, the PDE5 enzyme acts as the “biological timer” for erections by breaking down cyclic guanosine monophosphate (cGMP), the second messenger involved in regulating blood flow in the penis. Therefore, inhibition of this action by the use of a PDE5 inhibitor (PDE5i) results in a temporary vasodilatory action, which is clinically translated into recovery and/or improvement of erectile function and hardness. Following the introduction of sildenafil, other PDE5is with different pharmacodynamic and pharmacokinetic properties have been developed and approved for use in humans [2].

PDE5is are undeniably effective in most cases, and their safety profile is generally favorable [2]. However, when clinicians prescribe these drugs, it is not uncommon for patients to reply with a look of disbelief mixed with fear, followed by the same question: “*Is this treatment risky?*”. Likewise, clinicians who lack specific training in sexual medicine [3] might fail to understand the importance of sexual health – and, consequently, of sexual dysfunctions and their treatments – and might therefore miss the opportunity to investigate and treat conditions that unquestionably impair quality of life.

In the present review, we aimed to collect evidence of the cardiovascular (CV) effects of PDE5is to provide an up-to-date overview of the possible link between CV health and sexual function and to highlight the potential uses of PDE5is outside of their main intended scope to further demonstrate their safety profile related to CV health.

### Pathophysiology of erectile dysfunction and mechanisms of action of PDE5 inhibitors

**Fig. 1:**
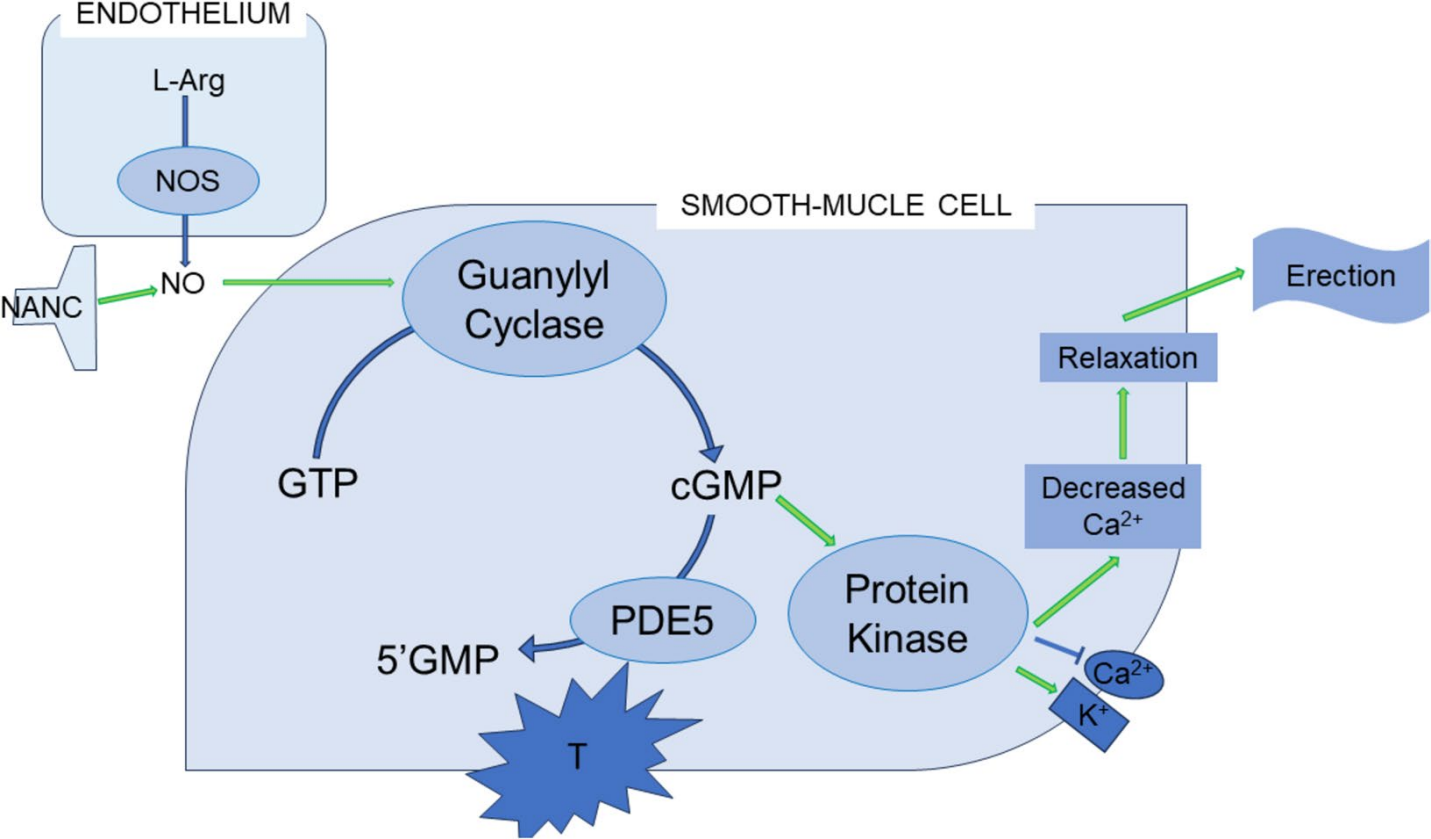
Biochemical pathways promoting erection. Abbreviations: NOS: nitric oxide synthase; L-Arg: L-arginine; NO: nitric oxide; SMC: smooth muscle cell; GTP: guanosine triphosphate; cGMP: cyclic guanosine monophosphate; K: potassium; Ca: calcium; T: testosterone; PDE5: type 5 phosphodiesterase

ED is one of the most common male sexual dysfunctions, resulting in the inability to obtain and/or maintain an erection adequate for sexual activity. ED can occur in the presence of several systemic conditions, but it is often the result of several risk factors – organic, intrapsychic, relational, and iatrogenic – affecting an individual at a given time [2]. Some background information on the physiology of erection is necessary to better understand how ED can develop. In the presence of sexual stimuli, erection occurs in a healthy man following a very complex network of systems [4]: the sexual stimuli are received from the brain and transmitted to the spinal cord, where they modulate the spinal erection centers at T11–L2 (promoting psychogenic erection) and S2–S4 (promoting mechanical, or reflexogenic, erection) [5]. Cholinergic and nonadrenergic, noncholinergic (NANC) nerve fibers of the corpora cavernosa therefore release neurotransmitters with vasodilatory properties, among which the most important molecule is nitric oxide (NO). NO is produced by the NO synthase enzyme (NOS), which is mostly present in nerve terminations (neuronal NOS; nNOS) and in endothelial cells (endothelial NOS; eNOS): the reaction catalyzed by NOS involves arginine nicotinamide-adenine dinucleotide phosphate (NADPH), hydrogen and oxygen and generates NO, citrulline, NADP + and water molecules as byproducts [6]. NO acts at the intracellular level by activating soluble guanylate cyclase (sGC), an enzyme that catalyzes the production of cyclic GMP (cGMP) from GTP: cGMP acts as a second messenger by activating a protein kinase that ultimately triggers the opening of potassium channels, leading to hyperpolarization of the membrane of the muscle cell (Fig. 1). A subsequent chain of events results in decreased cytosolic calcium levels and relaxation of vascular smooth muscle, which promote vasodilation. Given the anatomical structure of the penis, vasodilation causes tumescence, compressing the penile veins against the tunica albuginea and therefore increasing blood pressure in the penis [7].

Several factors can contribute to the development of ED, or its progression from subclinical to clinically overt, more severe forms [8]. First and foremost, ED is a consequence of impaired blood flow in the penis, resulting from reduced inflow, increased outflow, or both [4]. It is textbook knowledge that all lifestyle factors that can have detrimental effects on vascular parameters, or to a broader extent, on general health – smoking, malnutrition, lack of physical exercise, and alcohol abuse – can affect erectile function [9]. Acting on these risk factors is therefore suggested as a first-line therapy: this includes smoking cessation, or at least switching to modified risk tobacco products (heat-not-burn devices) [10]; the use of healthy dietary habits, such as the Mediterranean [11] or ketogenic diet [12]; and moderate physical activity [13]. Metabolic factors also play a role in the development of ED [9]: diabetes mellitus is undoubtedly the most prevalent factor [14, 15], but obesity and dyslipidemia, both highly prevalent conditions in the general population, are also associated with ED [16–18]. Many other conditions have been studied as additional risk factors for ED, such as hyperhomocysteinemia [19, 20], hyperuricemia and gout [21, 22], short- and long-term complications of COVID-19 [23, 24], and neurological diseases [25]. CV diseases (CVDs) are commonly associated with ED and share the same risk factors; that is, hypertension and dyslipidemia, as well as chronic obstructive pulmonary disease and other CVDs, all of which increase the risk of developing ED [4, 7, 17]. Unsurprisingly, most medications used to treat hypertension also worsen erectile function since they affect the hemodynamics of the penile vascular bed [2, 4].

Countless additional factors are involved in the pathogenesis of ED. We believe that some discussion is necessary regarding the conditions affecting sexual desire and arousal. These two parts of the sexual response cycle can be reduced in patients with several endocrine conditions, such as hypogonadism, thyroid disorders and hyperprolactinemia [26]. Testosterone is also necessary for adequate transmission of sexual stimuli and modulates the male sexual response through other pathways: some studies in animal models of hypogonadism have reported a rapid decrease in the expression of nNOS [27], and others have described how α1-adrenergic responsiveness is largely dependent on androgen levels in smooth muscle cells [28]. Some studies have also suggested that androgen levels might upregulate the expression of PDE5 [29, 30], although this hypothesis has been questioned as being counterintuitive in that increased androgen levels would impair the duration of erection, with the reduced PDE5 levels associated with a decline in smooth muscle cells reported in hypogonadal men [31]. In addition to endocrine dysfunctions, several other factors can lead to reduced desire and arousal, such as depression [32, 33], anxiety [34], and other psychiatric conditions [35], contributing to increased rates of ED. Psychological and relational issues, such as infertility [36, 37], sexual desire discrepancy [38], and the presence of comorbid sexual dysfunctions in the patient (premature ejaculation, “lost penis syndrome”, reduced penile sensitivity) [39–41] or in the partner (hypoactive sexual desire disorder, vaginismus, meno- and couplepause) [42–45], can similarly affect the ability of a man to obtain or maintain an adequate erection. In these regards, it would conceptually be wrong to define ED as “organic vs. psychogenic” [46], as, in fact, all sexual dysfunctions can lead to psychological or relational issues, and there is no way to prove that some forms of ED are purely *mind-generated* (the literal translation of psychogenic) [2].

Despite the plethora of factors possibly affecting erection, PDE5is have shown reliable efficacy in most forms of ED, despite treatment failures occurring in some individuals (see below). PDE5 acts as a “biological timer” for erection by hydrolyzing cGMP to GMP, therefore shutting off the biochemical mechanisms that allow vasodilation of the penile vessels [47]. By inhibiting the PDE5 enzyme, the hydrolysis of cGMP to GMP is reduced; therefore, the subsequent chain of events resulting in proerectile changes is enhanced. As these mechanisms depend on sexual desire, PDE5is do not induce erection without sexual stimuli.

### History of PDE5i

While this would seem completely unacceptable from a 2024-and-beyond perspective, up to the last decades of the 20th century, ED was considered a “minor” nuisance, mostly depending on psychological issues [48]. Since many men failed to improve their erection despite psychological support, it soon became apparent that ED could also be the result of additional underlying organic factors. Scientific interest in the diagnostic and therapeutic possibilities for ED grew exponentially during the 1970s and 1980s, famously ending with Dr. Giles Brindley’s intracavernosal self-administration of phenoxybenzamine just before going on stage at the American Urological Association (AUA) in 1983 [49]. The AUA lecture by Brindley highlighted the possibility of managing ED with efficacious, although intrusive, treatments, paving the way towards the development of novel treatments for ED.

Between 1991 and 1992, three groups of researchers (Jacob Rajfer and Louis Ignarro; Noel Kim, Inigo Saenz de Tejada, and Irwin Goldstein; Arthur Burnett and Solomon Snyder) identified NO as the molecule involved in erection [50–53]. At approximately the same time, the first “modern” definition of ED was proposed by the NIH Consensus Panel on Impotence [54]. However, despite growing public and scientific interest in ED, valid treatment was still not available.

As described in several books and papers [55, 56], the discovery of sildenafil was serendipitous, occurring while researchers at Pfizer were investigating drugs for the treatment of angina. Since PDE5 had already been identified in platelets and smooth muscle cells [57, 58], researchers postulated that its inhibition would yield beneficial vasodilatory effects; clinical trials later reported transient and limited efficacy from a CV perspective [59]. Additionally, such drugs had a brief half-life, and some studies reported some minor but concerning side effects, such as muscle pain and a blue tinge in the field of vision (cyanopsia), rarely present at the highest doses. Interestingly, researchers also noted the presence of an increased frequency of penile erections among side effects. This led to a previously unexpected shift in interest in PDE5is; their effect on CV parameters was deemed not worthy of the efforts of subsequent clinical trials, whereas the possibility of developing the first oral drug for the treatment of ED quickly became a reality. In the end, an otherwise minor drug that would have yielded only marginal benefits for CVD was “converted” to a groundbreaking treatment for ED, as subsequently proven by several trials that proved beyond a shadow of doubt the reliability of sildenafil as a first-line oral treatment.

### Safety of PDE5is in cardiovascular disease

When PDE5is are prescribed, it is not uncommon for patients to be worried concerning the potential risk of these drugs for CV health. This is somewhat surprising, considering the history of sildenafil; however, anecdotal reports of myocardial infarction in men using proerectile drugs, mostly published in newspapers rather than in peer-reviewed journals, have led many people to question whether the use of sildenafil or any other PDE5i would result in a greater likelihood of developing serious CV complications. Over the last 25 years, several authors have investigated this possible association, and current evidence seems to suggest that PDE5is are safe for use in *most*, but not all, patients with CVD.

**Fig. 2:**
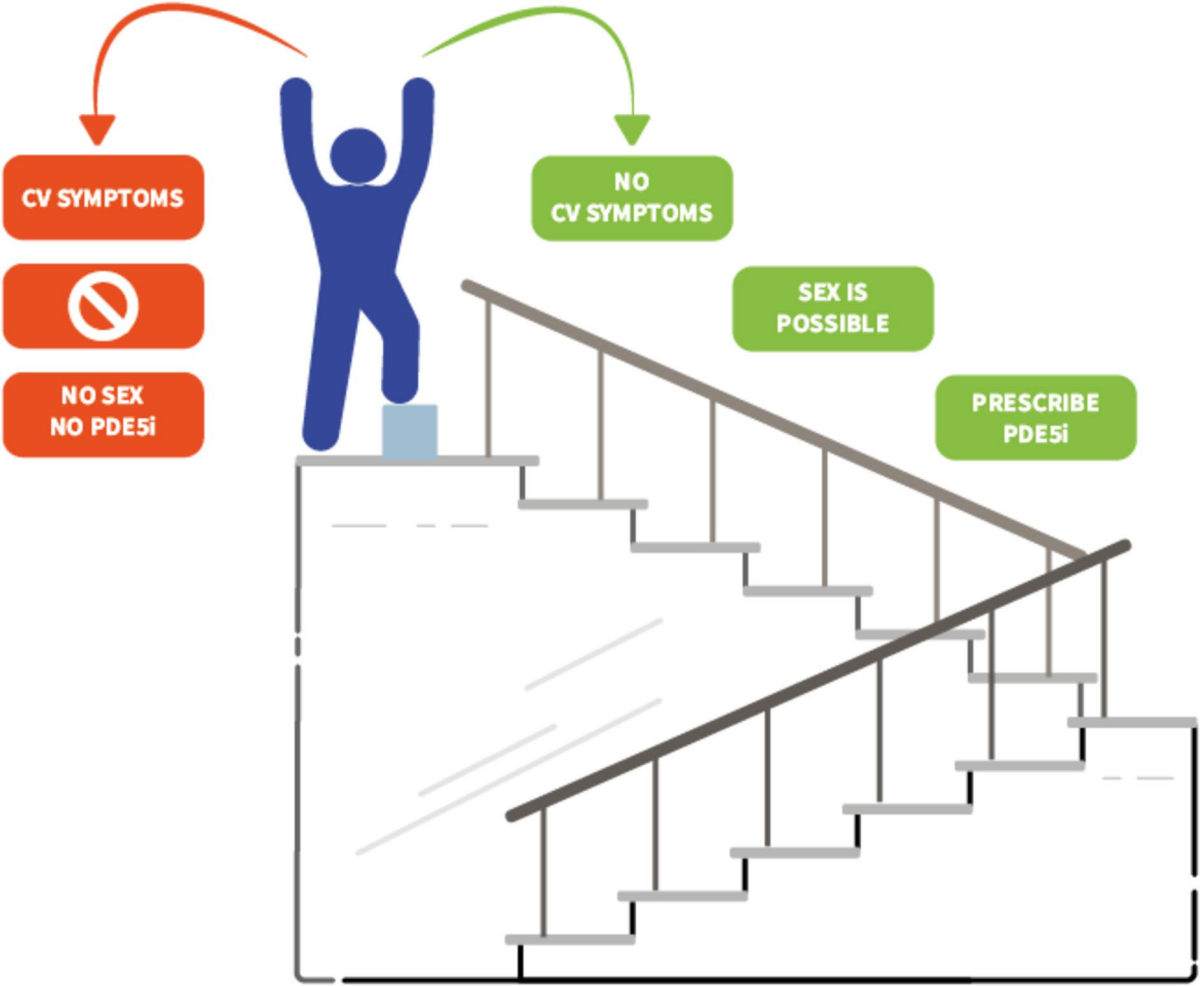
The rule of climbing 2 flights of stairs. According to the Princeton Conference [55, 56], cardiovascular risk may be clinically apparent when a patient cannot climb 2 flights of stairs. In this case, sexual intercourse (and thus the PDE5i assumption) should be discouraged. Abbreviations: CV: cardiovascular; PDE5i: type 5 phosphodiesterase inhibitor

To gain a better understanding of the safety and risks of PDE5i use in these patients, it is necessary to consider several fundamental premises. First and foremost, CVDs are highly prevalent in the general population and share the same risk factors as ED [9]. One of the leading hypotheses suggests that ED can be an early marker of CVD owing to the smaller caliber of the penile arteries compared with that of the vessels involved in major acute CV events (MACE) [60]. Therefore, sexual dysfunction could be the proverbial canary in the coal mine [61, 62], becoming clinically evident by an average of 2–5 years before the development of major CV events. Second, sexual activity, either with or without the use of PDE5is, can be stressful for all partners involved. In this context, a consensus conference was first held in 1999 at Princeton to address the potential risks of sexual activity for CV health [63]; in 2023, the Princeton Consensus Conference was reconvened for the fourth time [64]. In almost 25 years, despite an ever-growing body of evidence, the conclusions of the experts involved in the different Princeton Consensus Conferences have not changed: 30 min of sexual activity would roughly correspond to 2 or 3 METS (metabolic equivalent of tasks), or, in other words, to the same workload of climbing 2 flights of stairs [64]. Hence, a patient who may experience any CV symptoms while climbing 2 flights of stairs should be advised not to engage in sexual activity [65]. To simplify the issue for clinical purposes, the major cardiovascular risk is not the PDE5i itself, but rather the sexual activity of at-risk patients (Fig. 2).

In summary, these findings suggest that men with ED might have an underlying, possibly *subclinical*, CV issue, which presents initially as ED owing to the co-presence of organic (vascular and endocrine) and nonorganic (psychological and relational) factors and that being involved in sexual activity can result in an increased risk of complications owing to the metabolic expenditure of sexual activity itself. This raises the question: if ED is more prevalent in men with poor CV health and if sexual activity is a potential risk factor for MACE, is treatment with a PDE5i potentially harmful to patients?

The inherent risk associated with sexual activity can be easily measured using different algorithms, such as the Framingham Risk Score or the Systematic Coronary Risk Estimation 2 and the Systematic Coronary Risk Estimation 2-Older Persons (SCORE) chart [2]. The Princeton 4 Consensus Conference also suggested the use of the Atherosclerotic Cardiovascular Disease (ASCVD) risk estimator to measure the risk of major CV events, as well as the measurement of coronary artery calcium for men between 40 and 60 years of age with borderline to intermediate risk scores and no known history of CVD [64]. Algorithms ultimately allow stratification of patients into three classes of risk. Men at high risk should be referred to a cardiologist before considering sexual activity; those at intermediate or indeterminate risk should undergo stress testing to measure exercise tolerance; and men at low risk may be suggested for treatment with PDE5is.

The use of this calculated CV risk has some merits as well as some flaws. While all algorithms can be helpful for assessing the overall risk of developing CVDs, thus predicting the likelihood of severe complications, lifestyle changes should be suggested to all patients [66], even those at low risk, to delay progression to more severe forms of ED [8] or to prevent the development of other sexual dysfunctions [39, 40]. For patients at high risk, sexual activity should be put on hold until the CV condition is treated until stabilization [64], after which, from the perspective of harm reduction [67], the use of short-acting PDE5is is suggested to reduce possible interactions with other drugs used for CV management [2].

The safety of PDE5is in CVD treatment also deserves attention in terms of possible drug interactions. Owing to their vasodilatory properties, PDE5is should not be administered to patients treated with nitrates (absolute contraindication) or α-blockers (relative contraindication) [68]. Nitrates are NO donors and therefore increase the intracellular level of cGMP by activation of soluble guanylate cyclase; PDE5is act downstream by inhibiting the degradation of cGMP into GMP. Patients using both nitrates and PDE5is can therefore expose themselves to high concentrations of cGMP, resulting in exaggerated vasodilation and, subsequently, hypotension. However, nitrates might be overused in clinical practice, and long-term treatment with nitrates can potentially *increase* the rate of acute coronary events [64]. Therefore, it is highly advisable to consider whether this treatment is truly necessary and, if feasible, to suggest PDE5is if nitrate discontinuation is feasible. The risk of hypotension is also relevant for α-blockers: while some α-blockers, such as tamsulosin and silodosin, are uroselective and predominantly bind to the α1A receptor in the prostate and bladder, other drugs, such as doxazosin and terazosin, also bind to the α1B receptor present in vessels [69]. If possible, the use of uroselective α-blockers could be a valid choice to reduce the incidence of this condition, but there are potential risks of hypotension in patients who are candidates for PDE5i treatment.

Finally, overdosing PDE5is, possibly hoping for increased efficacy, is potentially dangerous for users’ health. Some authors have reported cases of aortic aneurysm dissection following the abuse of PDE5is [70], a finding that fits perfectly with the high levels of PDE5 expression in the human aorta. This becomes progressively important when considering that people who buy counterfeit drugs online might incur the risk of overdosing the drug, either because of mislabeling or because they voluntarily assume more than one tablet to compensate for the reduced efficacy of a single pill [71].

### Cardiovascular risk reduction associated with PDE5i use

As stated above, sildenafil was first identified while investigating drugs to be used to treat angina pectoris. It is therefore unsurprising that over the 25 years that followed the introduction of sildenafil on the market, additional evidence has been collected showing the beneficial effects of PDE5 inhibition on CV health. The NO-cGMP pathway is activated during ischemic heart disease to induce cardioprotective effects [72] via different molecular processes, mostly independent of vasodilation but rather based on anti-apoptotic and anti-inflammatory mechanisms [73–75]. Since PDE5is ultimately act downstream of the NO-cGMP pathway by preventing the hydrolysis of cGMP to GMP, these treatments can potentially reduce CV risk. However, it is still a matter of debate whether these treatments can have clinical use or whether these results are limited to an experimental setting. On the other hand, there is increasing evidence concerning the effects of PDE5is on other vascular parameters, with possible applications in the clinic. Patients with peripheral artery disease, such as Raynaud’s phenomenon [76] or arterial claudication [77], have reported beneficial effects of sildenafil and vardenafil.

The potential uses of PDE5is in CV prevention do not end here. PDE5is have been investigated as potential treatments for heart failure for more than 15 years [78]. Heart failure is a complex condition with a multifactorial etiology that includes ischemic heart disease, chronic obstructive pulmonary disease, and hypertension. It can be broadly defined according to the left ventricle ejection fraction as heart failure with preserved (HFpEF) or reduced (HFrEF) ejection fraction. Benefits have been reported in several clinical trials conducted in patients with HFrEF [79–81], whereas similar results were not reported in patients with HFpEF [80, 82]. This possibly results from the increased expression of PDE5 occurring in more severe forms of heart failure [70]. Therefore, while the potential benefits deserve further investigation, at present, the possible use of PDE5is for the treatment of HF is largely dependent on the clinical phenotype of the patient.

Diabetes mellitus is another condition in which treatment with PDE5is has shown remarkable success in terms of scientific research and potential translation for clinical use. Considering the high mortality rate of CVD in diabetic patients, particularly those with type 2 diabetes mellitus (T2DM), the potential preventive role of PDE5is in these patients has drawn much attention. Studies in animal models and in humans have shown beneficial effects on different heart parameters in diabetic patients treated with PDE5is [83], including cardiac geometry remodeling [84–86], in patients with diabetic cardiomyopathy. Notably, T2DM is often associated with other metabolic complications, such as metabolic syndrome, arthritis, and chronic kidney disease [14]. Endothelial dysfunction, a clinical finding often present in these patients owing to impaired NO availability, can not only worsen erectile function [87] but also promote the development of atherosclerotic CVD [88]. Endothelial dysfunction is present in diabetic patients [89–91] and is one of the possible factors involved in the pathogenesis of CV complications in T2DM patients. Treatment with sildenafil improves endothelial function [92, 93] and can therefore be considered a further tool in the hands of clinicians managing the CV health of diabetic patients. While the evidence is still not enough to warrant the clinical use of sildenafil (or any other PDE5i) for the treatment of diabetic heart kinetics or as a first-line treatment for other CV complications in such patients, this new avenue of research looks promising, and new trials will hopefully allow the drafting of guidelines for clinical practice.

### Treatment failure and subsequent psychological impact

While PDE5is are able to dramatically improve the ability to obtain and maintain erection in most patients, the efficacy of these drugs, as with all drugs, is not absolute. As ED is a multifactorial condition resulting from organic, psychological, relational, and iatrogenic risk factors, it is unsurprising that there is no “miracle drug” able to restore erectile function in 100% of cases. In some cases, treatment failure can potentially worsen the underlying ED by “adding insult to injury”. We believe that an example might be necessary here. A common clinical case involved a ~ 65-year-old male with some predisposing risk factors, such as smoking and metabolic syndrome, who presented to a sexual medicine expert due to an increasingly severe and frequent loss of erection. The patient complained of difficulties in obtaining and/or maintaining erection for intercourse in the previous year. Additionally, he reported loss of spontaneous erection and loss of libido, which are among the hallmark symptoms of male hypogonadism [94–98]. However, a somewhat distracted or untrained physician might prescribe PDE5is without investigating the endocrine status of the patient, resulting in a failed diagnosis. Considering the role of testosterone in all aspects of sexual function [26, 95], PDE5i treatment will most likely fail. The patient will subsequently become increasingly distressed by his poor erection, leading to the development of other sexual dysfunctions [40], depressed mood [99] and further loss of libido [100]. The patient will thus enter a downward spiral, abandoning a virtuous lifestyle for subsequent depression, and thus worsening his CV health. To mitigate such a decline, the patient should be adequately assessed and treated by a dedicated team of specialists, which should also include trained psychosexologists and/or an expert in sexual medicine.

As stated above, the response to PDE5i treatment is largely dependent on the integrity of the neurological apparatus involved in erection and of the vascular endothelium, both of which are sources of NO (via nNOS and eNOS). In particular, sexual stimuli trigger the activation of nNOS, mediating the conversion from an electrical (neurological) to a biochemical (NO) response. In the absence of NO release or if the vascular endothelium is less responsive to NO stimulation, PDE5is are less effective, since cGMP levels do not increase, and therefore, there is less substrate upon which to exert their effects. Therefore, PDE5is do not induce erection in the absence of valid sexual stimuli, unlike other vasoactive agents (such as alprostadil). Likewise, the response is blunted in cases of severe nerve or vascular damage, such as following radical prostatectomy and/or pelvic radiation therapy [2, 4].

Another possible reason for treatment failure lies in the adherence to treatment. While some patients prefer short-acting, on-demand treatment, others might prefer either long-acting therapy or daily, low-dose treatment [2]. In principle, long-acting drugs should not be used if the patient is receiving treatment with potentially interfering medications (e.g., antihypertensive agents and antiretroviral therapy), while they are indicated – at low daily doses – in patients complaining of lower urinary tract symptoms (LUTS) [101]. Identifying which treatment would be more suitable for each patient can help reduce potential drop-outs, which, despite the efficacy of PDE5is, remain remarkably high [102]. The issue of treatment drop-out is particularly relevant for PDE5is, given that only a minority of patients with ED actually seek medical treatment [103] due to stigma, shame or simple lack of awareness. The presence of a supportive partner can have beneficial effects on treatment compliance [104], as well as on CV and overall health [105, 106]. In an open trial comparing the efficacy of different PDE5is, sildenafil, tadalafil and vardenafil had similar effects on a subjective scale, as post-treatment scores of the short International Index of Erectile Function Questionnaire [107] did not differ across different treatment groups [108]; however, sildenafil showed better objective results when measuring penile hemodynamics via color-Doppler ultrasound, suggesting significant improvements in endothelial function [108].

Finally, the black market of counterfeited drugs has flourished enormously in the last few years. PDE5i are among the most counterfeit products worldwide [71] since, in many cases, people prefer to buy such drugs anonymously to avoid shame and embarrassment. However, counterfeit PDE5i pose several risks to unaware users, such as varying dosages of the active pharmaceutical ingredients and adulteration with other drugs or toxic compounds [71]. Additionally, buying drugs online without any prior consultation can potentially expose patients to drug interactions and reduce the rate of diagnosis for all those conditions – diabetes, CVDs, endocrine disorders – which can result in the development or progression of ED [71, 109, 110].

### Addressing an unmet need: choosing the right dosage for each patient

Sildenafil was originally developed and marketed at three dosages, 25, 50, and 100 mg, with a dosage of 50 mg being suggested as the starting dose for patients naïve to treatment [111]. The 100 mg dosage has therefore long been considered the only viable alternative for patients who respond to sildenafil but are not fully satisfied by the increase in erection hardness and duration under the 50 mg dosage. However, for virtually all drugs, a higher dosage of the drug can potentially expose the users to increasingly severe side effects. As previously described, the side effects of PDE5is are generally tolerable and short-term; however, from a bioethical perspective from centuries ago, clinicians should always consider the Latin maxim *Primum non nocere* (“firstly, do no harm”). Sexual medicine experts could find themselves in difficult situations in which a patient might not be satisfied with the starting recommended dosage (sildenafil 50 mg, vardenafil 10 mg, tadalafil 10 mg, avanafil 100 mg – Table 1), but the same patient would not be able to tolerate the possible side effects of the highest dosage (sildenafil 100 mg, vardenafil 20 mg, tadalafil 20 mg, avanafil 200 mg). This clinical scenario highlights one of several possible reasons for drop-out from PDE5is [102]: perceived efficacy and side effects are both dose dependent, and as such, the delicate balance between treatment efficacy and safety rests on the clinician’s decision. The introduction of an intermediate dosage – 75 mg – represents an additional tool in the hands of the sexual medicine expert [112], as it can provide a tailored solution to the scenario described above.

**Table 1:** The four commercially available PDE5is, their dosages, formulations, pharmacodynamic and pharmacokinetic properties, and summary of evidence for cardiovascular (CV) health. Adapted from [2]. Abbreviations: FCT, film-coated tablet; ODT, orodispersible tablet; ODF, orodispersible film; t max, peak plasma time; t1/2, elimination half-life; IC50, half maximal inhibitory concentration

	Sildenafil	Vardenafil	Tadalafil	Avanafil
Approved in	1998	2003	2003	2012
Currently available dosages (mg)	25, 50, 75, 100	5, 10, 20	5, 10, 20	50,100, 200
Currently available formulations	FCT, ODT, ODF	FCT, ODT	FCT	FCT
Starting recommended dose	50 mg	10 mg	10 mg	100 mg
t max (hours)	~ 1	0.66–1.5	2	0.5–0.75
Duration of action (hours)	12	12	36	6
t 1/2 (hours)	3–5	4	17.5	3–5
IC50 (nmol/L)	1.6–3.9	0.1–0.7	0.94–4.0	5.2
Mode of use	on demand	on demand	on demand (10–20 mg), daily (5 mg)	on demand
Evidence for CV health benefits	Yes [79, 80, 84, 85, 92, 93, 118]	Yes [118]	Yes [86, 118]	Not available

The dosage is currently only available as an orodispersible film (ODF), whose ease of use is an added value for this formulation, which can increase adherence to the medication [113–115]. The ODF can be taken orally by the patient even without water and can be kept in a pocket or wallet owing to greater resistance to physical deformation, making this treatment more intimacy sparing [115, 116]. Additionally, the ODF is virtually the only formulation exempt from the risk of counterfeiting [71, 112].

The 75 mg dosage thus represents a valid alternative to the usually recommended 50 mg starting dose, as well as a potential solution to “rescue” patients who would otherwise discontinue treatment because of either lower efficacy (using a lower dose) or more frequent side effects (using a higher dose). Moreover, clinical experience suggests that the intermediate dose of 75 mg is close to the clinical efficacy of the 100 mg highest dose, with a safety profile of the entry dose of 50 mg [112].

### Looking to the future: evidence of the beneficial effects of PDE5is in CV diseases

The vasodilatory properties of sildenafil—and, by extension, of all PDE5is—are the main reason for its efficacy. Following its introduction on the market, several researchers have identified new potential targets for PDE5 inhibition. PDE5i can induce vasodilation in vascular beds other than the penile arteries, and new therapeutic uses have been identified [75].

Data from clinical trials suggest beneficial effects of PDE5is in terms of survival and clinical improvement for patients with pulmonary hypertension [117]. A recent meta-analysis confirmed these findings [118]; however, these findings only apply to the subgroup of patients with pulmonary artery hypertension, whereas there is less solid evidence in other subgroups, such as pulmonary hypertension secondary to left-heart disease, lung disease, or thrombotic obstruction [118].

Platelets are another possible target for PDE5is. By increasing platelet sensitivity to NO via the cGMP-dependent protein kinase pathway, sildenafil can prevent platelet aggregation [119, 120]. In rat models, the same pathway was found to be associated with neointimal hyperplasia, and treatment with sildenafil resulted in the inhibition of neointimal formation [120].

Overall, several studies suggest that PDE5is improve systemic endothelial function [121] and can have beneficial cardioprotective effects. For example, in a study published in 2017 by Andersson et al. on more than 43,000 Swedish men hospitalized for myocardial infarction (mean age 64 ± 10 years), mortality was 33% lower among the more than 3000 (7.1%) men who had received treatment with PDE5i or alprostadil than among the untreated group [122]. Moreover, lower mortality rates were reported by Vestergaard et al. (treated: *n* > 71,000, mean age 60.7 ± 8.4 years) [123] and Kloner et al. (treated: *n* > 23,800, mean age 51.7 ± 10.4 years) [124], suggesting a dose-dependent effect, with the lowest incidence of MACE for men in the highest quartile of PDE5i exposure. While these studies agree on the main results, they are all retrospective studies, and as such, their reliability is somewhat limited: not only does the association not imply causation, but several other factors, such as increased awareness of the link between sexual function and CV health or preventive effects of sexual activity on CV health, could act as possible confounding factors.

## Conclusions

PDE5i were discovered serendipitously, but this fortuitous event drastically changed the medical management of ED. PDE5is quickly rose to the top of the most famous brand names all times, partly because of media attention, but mostly because they are undeniably effective. Despite over 25 years of evidence suggesting a potential beneficial role for PDE5is in the treatment of CVDs, it is not uncommon for patients to be concerned about potential risks. However, there is undisputable evidence of the beneficial effects of PDE5is on CV health. The most recent introduction on the market of PDE5is is the 75 mg sildenafil ODF formulation, which provides an intermediate tailored treatment to patients to reduce potential side effects while increasing treatment efficacy. Overall, PDE5is are becoming increasingly useful in the medical treatment of several CV-related conditions, among which ED is the best known, and new studies will hopefully allow clinicians to identify new applications for these drugs.

## Data Availability

Data sharing is not applicable to this article as no datasets were generated or analysed during the current study.

## Abbreviations

PDE5: Phosphodiesterase 5
PDE5i: PDE5 inhibitor
ED: Erectile dysfunction
CVD: Cardiovascular disease
HCPs: Health care providers
cGMP: Cyclic guanosine monophosphate
CV: Cardiovascular
NANC: Non-adrenergic, non-cholinergic
NO: Nitric oxide
NOS: NO synthase
nNOS: Neuronal NOS
eNOS: Endothelial NOS
NADPH: Nicotinamide- adenine dinucleotide phosphate
sGC: Soluble guanylate cyclase
cGMP: cyclic GMP
CVDs: CV diseases
AUA: American Urological Association
MACE: Major acute CV events
METS: Metabolic equivalent of task
HFpEF: Heart failure with preserved ejection fraction
HFrEF: Heart failure with reduced ejection fraction
T2DM: Type 2 Diabetes Mellitus
LUTS: Lower urinary tract symptoms
ODF: Orodispersible film
